# Anti-parasitic activity of pelleted sainfoin (*Onobrychis viciifolia*) against *Ostertagia ostertagi* and *Cooperia oncophora* in calves

**DOI:** 10.1186/s13071-016-1617-z

**Published:** 2016-06-10

**Authors:** Olivier Desrues, Miguel Peña-Espinoza, Tina V. A. Hansen, Heidi L. Enemark, Stig M. Thamsborg

**Affiliations:** Parasitology and Aquatic Diseases, Department of Veterinary Disease Biology, University of Copenhagen, Dyrlægevej 100, DK-1870 Frederiksberg C, Denmark; Section of Bacteriology, Pathology and Parasitology, National Veterinary Institute, Technical University of Denmark, Bülowsvej 27, DK-1870 Frederiksberg C, Denmark; Norwegian Veterinary Institute, PO Box 750, Sentrum, N-0106 Norway

**Keywords:** Sainfoin, Condensed tannins, Nematodes, *Ostertagia ostertagi*, *Cooperia oncophora*, Cattle

## Abstract

**Background:**

Increasing anthelmintic-resistance in nematodes of ruminants emphasises the need for sustainable parasite control. Condensed tannin-containing legume forages such as sainfoin (*Onobrychis viciifolia*) have shown promising anthelmintic properties in small ruminants but this has never been explored in cattle. Therefore, our aim was to examine the efficacy of sainfoin against cattle nematodes in vivo.

**Methods:**

Fifteen Jersey male calves (2–4 month-old) were allocated into two groups and fed isoproteic and isoenergetic diets mainly composed of sainfoin pellets (Group SF; *n* = 9, three pens) or concentrate and grass-clover hay (Group CO; *n* = 6, two pens). After 16 days of adaptation, all animals were experimentally infected with 10,000 and 66,000 third-stage larvae of *Ostertagia ostertagi* and *Cooperia oncophora*, respectively. Egg excretion, blood parameters and bodyweights were recorded throughout the study. Worms were harvested by sieving for quantification and scanning electron microscopy (SEM) 42 days post-infection (dpi) when the calves were necropsied.

**Results:**

The number of *O. ostertagi* adults in the abomasum was reduced by 50 % in Group SF compared with Group CO (*P* < 0.05). This was further reflected in higher albumin (*P* < 0.1) and lower pepsinogen levels (*P* < 0.05) in Group SF at 21 dpi, and structural damage of the worm cuticle could be visualised by SEM. Yet, the nematode egg excretion in Group SF was not significantly different from that of the controls (*P* > 0.05). Likewise, no statistical difference in total worm burdens of *C. oncophora* was found between the groups. Weight gains were lower for Group SF (*P* < 0.05), which may reflect lower digestibility and phosphorus levels in the SF diet, despite similar feed intake at pen-level.

**Conclusions:**

Overall, the effect of sainfoin on abomasal nematodes corroborates results from studies with small ruminants and encourages further investigations of the use of this crop for control of cattle nematodes.

## Background

Gastrointestinal nematode (GIN) infections in grazing ruminant livestock remain an important problem, which affect health and welfare of the animals and cause serious economic losses. While anthelmintic (AH) drugs are still considered a keystone for GIN control in ruminants, the awareness of the impact of drug resistance among nematode populations [1] urges farmers and researchers worldwide to explore new strategies, including feeding of bioactive forages containing plant secondary metabolites (PSM) with AH properties. While PSM can be administered on a short term as herbal remedies, we opted for a “nutraceutical” approach, which also consider bioactive plants for their nutritive value and require long term consumption to obtain substantial health benefits, including AH effects [2]. Plants with condensed tannins (CT; syn. proanthocyanidins) have been thoroughly investigated in small ruminants, particularly the use of CT-rich temperate/subtropical leguminous forages: sulla (*Hedysarum coronarium*), lotus species (*Lotus corniculatus* and *L. pedunculatus)*, sericea lespedeza (*Lespedeza cuneata*) and sainfoin (*Onobrychis viciifolia*). Due to their nitrogen fixating capacity, legumes have an essential role to play for the sustainability and competitiveness of grassland-livestock systems in Europe [3].

CT are polyphenolic PSMs found throughout the plant kingdom resulting from the polymerisation of flavan-3-ols units; using either epi-/catechin, known as procyanidins (PC), or epi-/gallocatechin, known as prodelphinidins (PD). Both PC and PD have the ability to reversibly bind proteins and other macromolecules. They have shown beneficial effects on animal health through lower GIN parasitism and bloat prevention, on animal nutrition due to better use of dietary protein and by reducing the environmental impact of livestock by mitigating emissions of greenhouse gases [4]. However, the bioactivity of CT in vivo against GIN of small ruminants is known to be highly variable depending on the plant source, the parasite and the host [5]. Plants have different concentration and composition of CT according to species, cultivar, season and general growth conditions. Also, it seems that a CT concentration of 3–4 % of dry matter (DM) in plant material is a lower threshold for AH effects [5], albeit different methods were used to estimate CT and may give variable results. Moreover, CT with a higher proportion of PD units have been associated with a higher efficacy both in vivo and in vitro [6–10]. Forage preservation processes, e.g. ensiling [11] or pelleting, releasing CT from the plant cells, have been shown to alter the status of CT from “free” to “bound”, notably to proteins, but may also positively influence the AH activity. Moreover, parasite species and stages have different susceptibilities to CT [12–14]. In addition, different host species are differently adapted to CT and/or various gut environments ensuring the formation or release of CT-protein complexes [5]. This has been illustrated, e.g. in sheep and goats using wattle tannin [15]. Although most in vitro assays and short-term CT feeding trials have demonstrated a direct AH effect of CT [5], the high nutritive value of some temperate legumes and the protection of protein by CT from microbial degradation in the rumen (rumen by-pass protein) can improve the immune response and be indirectly detrimental to the parasites [16, 17].

To the best of our knowledge no data are available on potential AH effects of CT against cattle nematodes in vivo. However, sainfoin seems to be a good candidate based on in vitro studies [8, 18]. It is a crop known for its appealing features such as high palatability, high protein levels and importance in biodiversity preservation. In GIN infected small ruminants intake of sainfoin has been consistently associated with a reduction in faecal egg counts (FEC), except when CT levels were lower than 2 % of DM intake [19–23]. Several studies have indicated reduction in worm burdens at CT dietary concentrations of 5.1 % and 8 %, without negative effects on animal performance [21, 22].

Our main objective was to assess whether feeding sainfoin has an effect on pathophysiological and parasitological measurements in calves experimentally infected with the most pathogenic and prevalent GIN of cattle in temperate regions: *Ostertagia ostertagi* and *Cooperia oncophora*.

## Methods

### Animals

Fifteen Jersey male calves with no grazing history (2.5−4.5 month-old, 91.4 ± 24.0 kg [mean ± standard deviation, SD]) were acquired from a commercial farm. Upon arrival they were orally treated with fenbendazole (Panacur®Vet 10 %, MSD Animal Health, 5 mg/kg bodyweight [BW]) to ensure helminth-free condition (later confirmed with negative FEC one week post treatment). During the whole study period the calves were reared indoor in solid concrete floored pens with straw bedding and external feeders. All animals were allowed an acclimatisation period of 16 days and were clinically assessed on a daily basis throughout the study.

### Feed constituents and nutritional analysis

Sainfoin (*O. viciifolia*) pellets were provided by Multifolia/MG2MIX (Viâpres-le-Petit, France) Ltd., and consisted of a pure-stand 3^rd^ cut (cultivar: Perly) harvested in 2012 and processed to obtain dehydrated pellets. The other feed sources included grass-clover hay and a commercial concentrate (Danish Ragna Grønmix^©^, Danish Agro Ltd., Karise, DK). The level of DM, crude protein (CP), neutral detergent fibre (NDF) and ash was analysed in all feed components by an accredited, authorised laboratory (Eurofins Steins Laboratorium Ltd., Holstebro, DK). Digestible organic matter (DOM) was calculated considering OM content in feeds and OM digestibility (OMD) of hay, sainfoin and concentrate that was analysed by near-infrared (NIR) spectroscopy, an in vitro method [24] and in vitro enzymatic method (EFOS [25]), respectively. The values for OMD of sainfoin and concentrate were corrected to in vivo OMD (OMD %) using the following regression functions: OMD %_sainfoin_ = 4.1 + (0.959 × in vitro digestibility_Tilley_ & _Terry_ %) (used for most forages) and OMD %_concentrate_ = 22.0 + (0.752 × in vitro digestibility_EFOS_ %) [25]. Metabolisable energy levels, expressed in MJ/kg of DM, were calculated from the net energy lactation values, which were provided by manufacturers or laboratory, and divided by 0.65 [26]. Mineral composition values were obtained from suppliers. Feeds were analysed for CT content by the acetone-butanol-HCl assay [27] with pure fraction of CT from dried sainfoin as standard. In addition, CT in sainfoin pellets were characterised after *in situ* thiolysis [28] and LC-MS analysis [29]. This gives information on the content and composition of CT: their mean degree of polymerisation (mDP)-values (i.e. size), percentages of PC versus PD type tannins, and *cis* versus *trans* flavan-3-ol subunits.

### Experimental infections

Infective third-stage larvae (L3) for inoculations were isolated from donor calves experimentally infected with *O. ostertagi* and *C. oncophora* following standard larval culture procedures (14 days at 21 °C). The larvae were stored at 13 °C for 3 months until evaluation of ensheathment and viability 1–3 days prior to inoculation. Hundred baermannised L3 were then morphologically differentiated [30], and a batch comprising 13 % *O. ostertagi* and 87 % *C. oncophora* was used. All calves (*n* = 15) were orally inoculated with a total dose of 10,000 L3 of *O. ostertagi* and 66,000 L3 of *C. oncophora* per calf, given as three sub-doses for three consecutive days.

### Experimental design, diets and sample collection

Sixteen days prior to the experimental infection the calves were randomly allocated into two groups after stratification by BW. The Sainfoin group (SF) (*n* = 9; 90.9 ± 28.1 kg) was allocated to three pens, each containing three animals with almost similar BWs in order to allow a better estimation of the feed consumption and to avoid bullying (i.e. a pen for the smaller, intermediate and larger calves). The Control group (CO) (*n* = 6; 92.1 ± 18.5 kg) was allocated to two pens of three calves. All calves were weighed weekly except for the last time point.

In Group SF sainfoin pellets were gradually offered in addition to hay until becoming the main feed component (> 90 %) after a period of 12 days. Calves from Group CO received commercial concentrate (ranging from 55 to 65 % of the diet) and hay of ryegrass-clover. The intakes of sainfoin pellets, hay and commercial pellets were calculated every day for each pen (feed offered - feed refusals). Four days prior to infection, the diets were formulated in order to fulfil requirements of CP and energy and to obtain similar levels adjusted for BW in both groups. Diet in Group SF was offered close to *ad libitum* and the diet in Group CO was adjusted with concentrate every second day to match the diet of Group SF, with a maximum of 2 and 3 kg of DM for small and larger animals, respectively. All animals had free access to water. Straw bedding consumption was observed in both groups but could not be recorded. Individual faecal samples were rectally collected once weekly during the pre-patency period and every 2–3 days from 14 days post-infection (dpi) onwards. Faecal egg counts (FEC) and larval cultures were prepared as described in the next section. Individual blood samples were collected by jugular venipuncture at weekly intervals, and the recovered serum was stored at -20 °C until use. At 40−42 dpi all calves were euthanized with captive bolt pistol followed by exsanguination.

### Serum and faecal samples analysis

Serum samples obtained from week 0 to 6 were analysed for pepsinogen as previously described [31] and for total protein (TP), albumin (ALB) and inorganic phosphate (IP) using an ADVIA1800 analyzer (Siemens). A more detailed biochemical profile analysis was performed at necropsy: alkaline phosphatase, alanine aminotransferase, total bilirubin, cholesterol, creatinine, creatinine kinase, iron, aspartate aminotransferase, urea, gamma-glutamyl transpeptidase, calcium, magnesium, sodium and potassium.

FEC were determined using a modified McMaster technique with a sensitivity of 5 eggs per gram faeces [32]. Faecal DM from each animal was determined during the patency period from 3 g subsample dried at 60 °C for 24 hours, and FEC were expressed as the number of eggs per gram of dried faeces (FECDM). Species-specific FECDM was estimated from larval cultures that were prepared at selected time points from pooled faecal samples (10 g of faeces per animal) for each pen, which were mixed with vermiculite and incubated for 13 days at 20–22 °C.

### Recovery and analyses of adult nematodes

Immediately after euthanasia, the abomasum and the whole small intestine from each animal were opened separately and washed with 5 and 10 l of 38 °C warm saline (0.9 % NaCl), respectively. For each organ, one subsample of 500 ml of washed digesta content (i.e. representing 10 % of the abomasum and 5 % of the small intestine contents) was passed through a 38 μm metallic sieve and retained material was stored in 70 % ethanol. Worm counts and sex determination were performed. Sex ratio was reported as the percentage of male worms in the total worm burden. Changes in sex ratio are a good indicator of worm expulsion for *C. oncophora* because expulsion initially includes male worms [33]. Worm fecundity was assessed by counting the number of eggs from 16 female worms of both nematode species per calf. These worms were recovered from individual animals and lysed in 200 μl of a 20 % household bleach solution. Eggs were enumerated 10 min after lysis for *O. ostertagi* and after 30 min for *C. oncophora* female worms. Finally, the cleaned mucosa of the abomasum from one animal in each group was scraped and digested in pepsin/hydrochloric acid for 2–3 hours [34]. Each digested mucosa was passed over a 38 μm sieve, and the retained material was examined for inhibited fourth-stage larvae.

### Scanning electron microscopy (SEM)

Adult *O. ostertagi* were examined by SEM. The worms were collected from randomly selected animals (CO: *n* = 2; SF: *n* = 4) after washing with tap water, and fixed in PBS with 2 % glutaraldehyde. They were merged per Group and post-fixed in 1 % OsO_4_ and 0.05 M K_3_FeCN_6_ in 0.12 M cacodylate buffer for 2 hours at room temperature and stirred. Then, the samples were rinsed with 0.15 M cacodylate buffer and distilled water, followed by dehydration in a gradient of ethanol concentrations in a critical point dryer. The worms were coated with 4 nm gold with a coating instrument (Leica EM ACE200) and loaded in a scanning electron microscope (Phillips XL 30 FEG) for visual analysis using Scandium software.

### Statistical analyses

The effects of feeding sainfoin were assessed by linear mixed-effects models fitted with maximum likelihood. BW, faecal DM and biochemical markers were analysed as repeated measures as described by [35]. These models included Pen as random effect and the fixed effects were Group and Time, and the interaction between Group and Time if significant. Baseline measurements were used as covariates for biochemical markers (week 0 value) and BW (initial BW). Pepsinogen data were log-transformed prior statistical analysis but presented as back-transformed means. The variance homogeneity between observations of the models was also tested. The residual plots were used to validate the final models. Likewise, statistical differences between groups in relation to: worm counts (for *O. ostertagi*), sex ratio, fecundity of female worms (i.e. number of eggs *in utero*), and additional blood parameters (42 dpi) were analysed by mixed effects models including post-hoc comparison of means by Tukey’s test, with Group as fixed effect and Pen as random effect. Counts data for adult worms of *C. oncophora* and FECDM were over-dispersed therefore we used generalised linear mixed-effects models for the negative binomial family with Group as fixed effect and Pen as random effect. Statistical analyses were carried out with R (version 3.2.0).

## Results

### Feed composition and diets

The CT content of sainfoin pellets was 65 g/kg DM with the acetone-butanol-HCl method and 19.6 g/kg DM with the thiolysis LC-MS. CT had a high proportion of PD (81 %) and *cis*-conformation (77 %) and a mDP of 11. No CT were found neither in hay nor in concentrate. CP was low in hay compared to sainfoin and concentrate (8.4, 17.2 and 20.4 % of DM, respectively) (Table 1). The feed intakes for the week of experimental infection were 2.89 ± 0.05 for Group SF *versus* 2.87 ± 0.12 for Group CO (mean of pens ± SD kg/100 kg of BW). The feed intake in both groups increased gradually during the first 3 weeks post-infection and then stabilised around 3.5 kg/100 kg of BW for the last three weeks (mean of pens ± SD on the last week: 3.53 ± 0.06 in Group SF *versus* 3.58 ± 0.19 in Group CO). Dietary intakes are presented in Table 1 as pooled means per feeding group during the experimental infection period. CP dietary intake was marginally lower in Group CO (0.52 ± 0.06 kg/100 kg of BW) compared to Group SF (0.54 ± 0.04) but the energy intake was similar in both groups. Yet, diet for Group CO had higher DOM (2.31 ± 0.22) and NDF (1.36 ± 0.10) compared with Group SF (DOM: 1.92 ± 0.19; NDF: 0.92 ± 0.14).

**Table 1:** Nutrient contents of experimental feeds and daily dietary intake

Variables	Experimental feed				Daily dietary intake (mean across all weeks) (kg/100 kg BW)	
	Pelleted sainfoin	Hay of grass/clover	Concentrate		Group SF	Group CO
DM (%)	90.0	86.5	89.6		3.28 ± 0.33	3.36 ± 0.29
Ash (% of DM)	9.0	4.9	7.0			
CP (% of DM)	17.2	8.4	20.4		0.54 ± 0.04	0.52 ± 0.06
NDF (% of DM)	26.0	57.7	28.6		0.92 ± 0.14	1.36 ± 0.10
DOM (% of DM)	58.7	56.1	77.6		1.92 ± 0.19	2.31 ± 0.22
ME (MJ/kg DM)	9.7	7.2	10.5	(MJ/% BW)	31.0 ± 2.74	30.5 ± 2.92
P (% of DM)	0.2	–	0.5			
Ca (% of DM)	2.1	–	0.7			
CT (% of DM)	6.5	0.0	0.0			

Calves experimentally infected with gastrointestinal nematodes were allocated into two groups: Group CO (controls) fed hay and concentrate and Group SF fed tannin-rich sainfoin pellets

*Abbreviations*: *ME* metabolisable energy expressed in mega joules (MJ), *BW* body weight, *CP* crude protein, *NDF* neutral detergent fibre, *DOM* digestible organic matter, *P* phosphorus, *Ca* calcium, *CT* condensed tannins

### Health and bodyweights

Overall, the general condition of the animals was good during the study period although the youngest animals in Group CO had soft faeces to mild diarrhoea for most of the study period. The faecal DM was significantly higher in Group SF (*F*_(1,3)_ = 17.48, *P* = 0.025) across the patency period (pooled means [± SD] in Groups SF and CO, respectively: 18.2 ± 0.8 % and 15.2 ± 1.1 %). Although the effect of Time was not significant (*P* > 0.05), a gradual decrease in faecal DM was observed in Group CO from 14 to 28 dpi (16.0 ± 2.8 % and 12.8 ± 4.3 %, respectively) and then increased until 42 dpi (16.9 ± 3.1 %), which was not seen in Group SF.

**Fig. 1:**
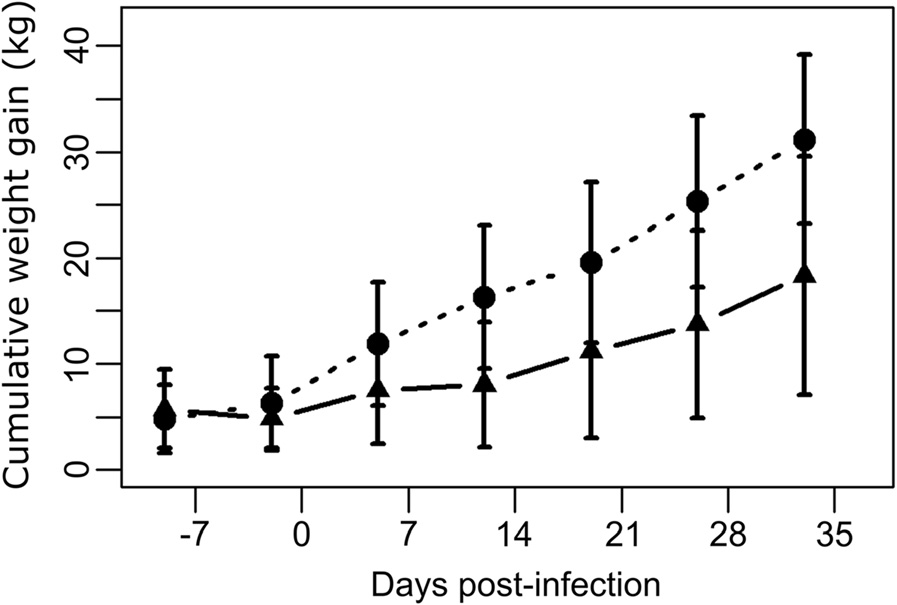
Cumulative weight gains (kg) of calves per group. Control group (CO; *n* = 6; *dotted line*) and Sainfoin group (SF; *n* = 9; *solid line*). Error bars represent the standard deviation. *P < 0.05

The BW of calves were significantly lower in Group SF as compared with Group CO from 5 dpi onwards (*P* < 0.05) with a significant interaction of Group × Time (*F*_(6,87)_ = 4.58, *P* = 0.0004). Moreover, the effect of the baseline was also significant (initial BW; *F*_(1,87)_ = 5189, *P* < 0.0001), as lower growth was observed in smaller animals. Cumulative weight gains (mean ± SD) for the whole period were 18.3 ± 11.3 and 31.2 ± 8.0 kg for Group SF and CO, respectively (Fig. 1).

### Blood parameters

**Fig. 2:**
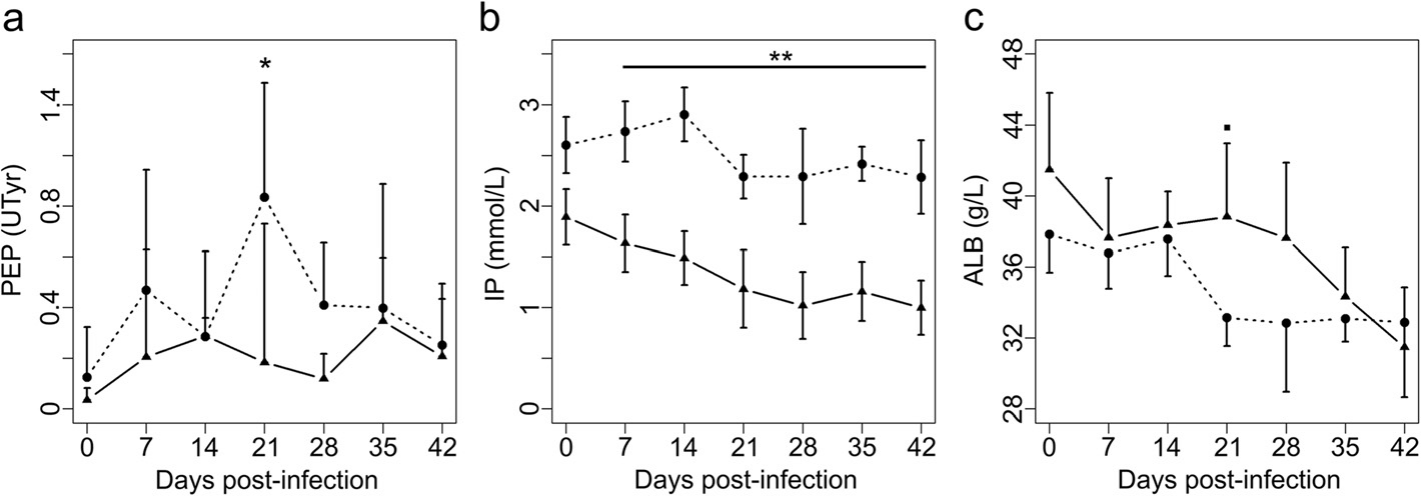
Mean serum levels of biomarkers in calves experimentally infected and fed various diets. **a** Pepsinogen (PEP). **b** Inorganic phosphate (IP). **c** Albumin (ALB). Control group (CO; *n* = 6; *dotted lines*) and Sainfoin group (SF; *n* = 9; *solid lines*). Error bars represent the standard deviation. ^▪^ *P* < 0.1; *P < 0.05; **P < 0.01

Serum PEP levels remained very low throughout the study in both groups (mean < 1 UTyr, Fig. 2a), indicating low infection levels with *O. ostertagi*. Nevertheless, the PEP levels were consistently lower in Group SF compared with Group CO, and significant differences (*t*_(3)_ = -3.74, *P* = 0.033) were observed 21 dpi. Serum IP values were significantly lower in Group SF (*F*_(1,3)_ = 71.14, *P* = 0.0035) throughout the study (Fig. 2b). Moreover, the baseline was not significant (*P* > 0.05) and the means in both groups seemed to level out after a drop around start of the patency period. Serum ALB levels decreased in both groups (Fig. 2c) but with significant effect of baseline (*F*_(1,74)_ = 6.06, *P* = 0.016) and interaction Group × Time (*F*_(5,74)_ = 3.27, *P* = 0.010); levels were higher in Group SF 21 dpi (*t*_(3)_ = 2.77, *P* = 0.069) as compared with Group CO. Serum protein levels gradually decreased during the study in both groups without any significant effect of Group (initial values: 71.2 ± 3.9; 68.0 ± 3.4 g/l; final values: 52.4 ± 4.4 and 55.7 ± 7.4 g/l for Groups SF and CO, respectively). Additionally, the levels of iron (20.2 ± 7.5 and 37.1 ± 9.6 μmol/l in Group SF and CO; GLH-test: *Z* = -3.84, *P* = 0.0001; Tukey) and gamma-glutamyl transpeptidase (8.9 ± 1.5 and 14.2 ± 3.7 U/l in Group SF and CO; GLH-test: *Z* = -3.94, *P* < 0.0001; Tukey) differed significantly between the groups.

### Faecal egg counts

**Fig. 3:**
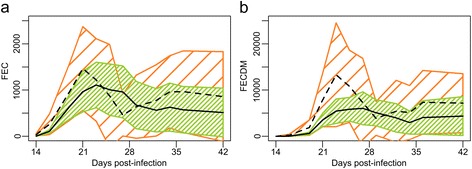
Mean faecal nematode egg counts during the patency period. **a** Faecal egg counts (FEC). **b** FEC adjusted for dry matter (FECDM). Control group (CO; dashed line: arithmetic mean; hatched orange area: 95 % confidence interval) and Sainfoin group (SF; solid line: arithmetic mean; hatched green area: 95 % confidence interval)

The means of FECDM were not significantly different between the groups (Fig. 3). Specific egg excretion of each nematode species was estimated from the pooled larval cultures, despite high individual variability of FECDM, and showed a similar trend as FECDM. Yet, an overall predominance of *C. oncophora* was found, particularly during the peak of excretion (mean % ± SD: 64 ± 6; 80 ± 15; 88 ± 10 and 86 ± 19 at 19, 22, 26 and 28 dpi, respectively), which was observed between 21 and 28 dpi for all calves except one in Group CO, in which it occurred around 35 dpi.

### Adult worms

**Fig. 4:**
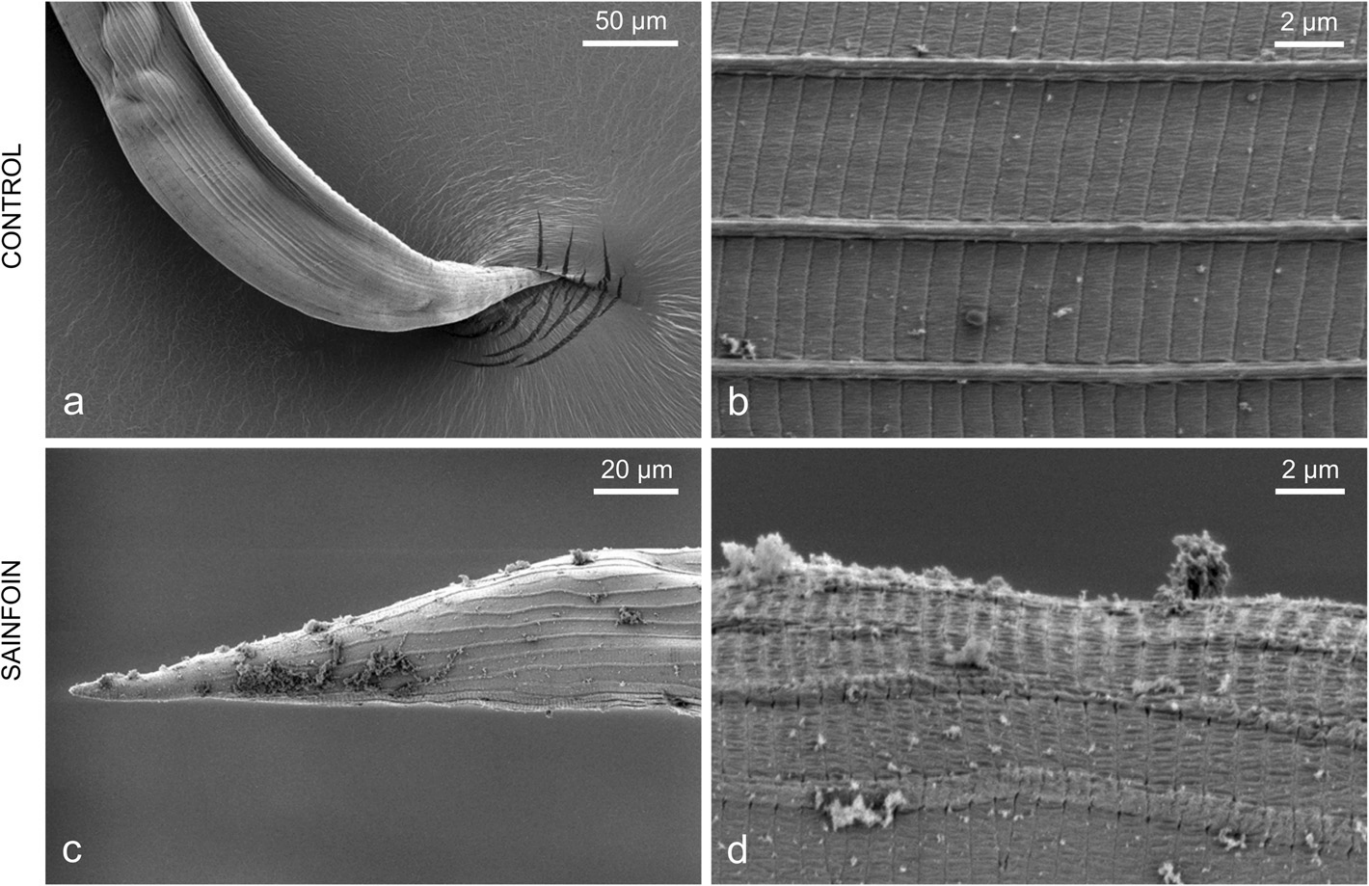
External structural changes of adult *Ostertagia ostertagi* recovered from calves fed sainfoin for 58 days. Scanning electron microscopy of representative worms recovered 42 days post-infection from Control group (CO) (**a**, **b**) or Sainfoin group (SF) (**c**, **d**). Left column: tail of the female worm; Right column: close view of the cuticle

The mean worm count of adult *O. ostertagi* was significantly lower in Group SF compared with Group CO (GLH-test: *Z* = -2.34, *P* = 0.019; Tukey; Table 2); a high worm burden (3,300) was found in the smallest animal in Group SF while all others had less than 1,500. The comparison of the statistical models with or without pen as a random effect was not significant with ANOVA. No significant differences were seen in sex ratio or fecundity of the female worms. Analysis of *O. ostertagi* adult worms by SEM (Fig. 4) showed some aggregates of particles and few localised damages of the external structure of 1/3 of the worms from Group SF (*n* = 3). In comparison, the cuticle of the worms from CO (*n* = 3) appeared clean and smooth (Fig. 4a, b). No inhibited fourth-stage larvae were recovered from the abomasal mucosa of the six animals.

**Table 2:** Parasitological data from worms recovered 42 days post-infection

Adult nematodes	Group	Worm burden	♂ (%)	♀ fecundity
*Ostertagia ostertagi*	CO	2,715 ± 894	44 ± 6	41 ± 09
	SF	1,331 ± 947^a^	42 ± 7	43 ± 12
*Cooperia oncophora*	CO	22,447 ± 17,639	34 ± 15	53 ± 45
	SF	19,664 ± 22,496	29 ± 23	40 ± 36

Data (mean ± SD) representing worm burden by sieving, percentage males and female fecundity based on the number of eggs *in utero* from 16 female worms per animal. The calves were experimentally infected with *Ostertagia ostertagi* and *Cooperia oncophora* and fed a tannin-rich diet (Group SF) or a control diet (Group CO)

^a^indicates a significant difference between the groups (GLH-test: *Z* = -2.34, *P* = 0.019; Tukey)

For *C. oncophora*, no significant differences between Group SF and CO (*P* > 0.05) were found regarding total worm burden, sex ratio and female fecundity. However, these results showed a strong variation within each group (Table 2). In Group SF, three animals (one in each pen) had close to none adult *C. oncophora* 42 dpi (≤ 100), and were therefore not included in the analysis of sex ratio and female fecundity.

## Discussion

This study showed that calves fed pelleted sainfoin as the sole, continuous diet had a significant reduction by 51 % in the worm burden of *O. ostertagi* 42 days after experimental inoculation, as compared with animals fed a control diet. Our results confirm AH effects of sainfoin observed in earlier studies of abomasal nematodes in sheep. In one study, sainfoin (6.1 % CT of dietary DM) consumption for 16 days significantly reduced the number of worms in an already established population of adult *H. contortus* [21]. Similar results have been reported for *Teladorsagia* spp. in naturally infected animals fed sainfoin containing 8.3 % CT for 13 days [22]. Moreover, the pelleting of sainfoin may have influenced the AH activity as shown by Terrill et al. [36]. These researchers found that consumption of the pelleted form of sericea lespedeza was even more potent as compared with hay of the same bioactive forage (both containing 6.4 % total CT) in reducing the worm burden of *H. contortus*. This was, however, not the case for *T. colubriformis*. Further, it has been highlighted that the molecular structure (e.g. PD/PC ratio) of CT may have a greater importance than the ratio of bound/unbound forms of CT in the diet [37]. This hypothesis was substantiated by our results. In fact, although bound CT were not determined directly in our study, the lower CT % obtained with thiolysis compared with acetone-butanol-HCl may indicate that CT have a low accessibility in the pellets. However, CT in sainfoin is mainly of the PD type and especially in our case (PD % > 80) that is known to greatly influence the AH activity of CT in vitro [8, 10].

Although the majority of feeding trials with sainfoin have been associated with a significant reduction in FEC, the non-significant egg count reduction in our study was presumably due to the apparent lack of effect on *C. oncophora* which was the dominant species in the inoculum and responsible for the majority of the egg output throughout the trial. A few studies found that a lower female worm fecundity, rather than a lower worm burden, was responsible for FEC reduction in goats and sheep [20, 23]. Other studies have reported reduced numbers of adult *H. contortus* in lambs without any accompanying significant effect on worm fecundity after feeding sainfoin for 16 or 70 days [21, 38].

We expect that the effect of sainfoin against *O. ostertagi* is mainly related to direct effects of CT rather than an immunologically induced expulsion, since it is known that the acquisition of protective immunity against *O. ostertagi* needs a continuous larval exposure for longer than the first grazing season to arise [39]. Moreover, it has been shown that the loss of adult worms was very limited even 50 dpi after a single infection with 10,000 L3 of *O. ostertagi* [40]. Our study was not designed to pinpoint which stage of infection was affected by sainfoin. The levels of serum-pepsinogen indicated a low infection level [41] and the albumin values were in all cases within the range of healthy calves [42], although significant differences were found between groups. However, the lower pepsinogen in Group SF at 21 dpi, when *O. ostertagi* young adults had emerged from the gastric glands, could indicate an effect of sainfoin at this stage of infection, or perhaps even on establishment. In fact, Brunet et al. [18] found that the larval exsheathment of *O. ostertagi* was reduced in vitro in the presence of sainfoin extracts. Furthermore, sainfoin has been shown to reduce the larval exsheathment process of *H. contortus* in the rumen of cannulated sheep fed with fresh sainfoin [43], and to inhibit the following penetration into the abomasal mucosa after in vitro incubation with sainfoin extracts [44]. The authors linked these effects with structural alterations of the infective larvae at both external and internal levels, depending on the presence of the sheath [45]. Moreover, we also found local cuticular damage by SEM on adult *O. ostertagi* of sainfoin-fed calves (Fig. 4). Previously, structural damage of worms at intestinal and muscular levels have been visualised by transmission electron microscopy of adult female *H. contortus* from goats fed sainfoin [46]. The external surfaces of worms from goats was also damaged when animals were fed sainfoin, tzalam (*Lysiloma latisiliquum*) [47] and more recently sericea lespedeza [48]. Interestingly, the latter study concluded that the degree of cuticle damage was influenced by the length of exposure to CT; all worms were affected after 77 treatment days whereas only a smaller proportion was affected after 28 days. In our case, adult worms were in contact with CT for approximately 25 days which may explain the inconsistencies between worms. In addition, adult worms were pooled per group and calves may have had different CT concentrations in the digestive tract considering the variation in straw consumption.

Our study showed no influence of sainfoin-feeding on the worm counts of *C. oncophora* in the small intestine. This is in accordance with a previous study of established populations of *C. curticei* in experimentally infected sheep fed sainfoin (6.1 % CT in DM) [21]. The same study reported a significant reduction of abomasal species. In addition, they found a significantly lower fecundity of *C. curticei*, although no data *in utero* were presented [21]. However, a reduction in numbers of adult *C. curticei* has been reported in naturally infected lambs fed sulla (*Hedysarum coronarium*) which had a high CT content (12 % CT of DM in the leaves) [49]. Despite these examples, there seems to be an apparent lack of bioactivity of CT in sainfoin against nematodes in the small intestine. This may be due to a lower availability or binding capacity in the small intestine where the local conditions are different from the abomasum [5]. Accordingly, the majority of studies in sheep have reported a greater effect of sainfoin against abomasal versus small intestinal nematodes [21, 38, 50], but similar AH effect of sainfoin in the two gut compartments has also been reported [23]. The opposite case: a better effect against small intestinal nematodes, was only reported in studies of sheep fed with quebracho as a source of CT that contains profisetinidins, another type of flavanol basic units [51]. Controlled in vivo and in vitro studies with tannins against cattle nematodes at species level are very limited. Recently, it has been reported that *O. ostertagi* was more susceptible to CT than *C. oncophora* in vitro [10], although such difference was not found in other studies [8, 18]. In vivo studies may yield a different result. The low number of adult *C. oncophora* recovered from some calves in both groups, 42 dpi, was likely related to the expulsion of worms in those animals. In fact, a primary infection with *C. oncophora* has been shown to elicit a Th2-biased immune response against adult worms in high responder type animals, which confers a rapid protective immunity [33]. Moreover, in the same study the worm sex ratio proved to be a good indicator of the worm expulsion as males were first expelled, which was also observed in our case.

In general, calves appeared healthy and showed no signs of clinical disease. Group SF and had less soft faeces compared with Group CO. Although we found higher levels of iron and gamma-glutamyl transpeptidase in serum of calves of Group CO compared with Group SF 42 dpi, only one value of serum iron in Group SF was slightly lower than the reference range for calves [42, 52]. Therefore we did not consider this biologically significant. However, the mean levels of inorganic phosphorus in Group SF were ≤ 1.3 mmol/l from 21 dpi which is at the lower marginal threshold for calves [53]. This emphasizes the need for possible supplementation with phosphorus. In previous studies, infected small ruminants fed sainfoin hay had similar or improved weight gain with CT concentrations up to 8 % [21–23]. Yet, the relatively low number and variable size of animals in our study preclude firm conclusions on performance. Moreover, it was challenging to maintain similar levels of CP and energy in the diets of CO and SF groups with the low replication of feed intake. Therefore the feed intake adjustments and nutritional differences in favour of the concentrate-rich diet in Group CO including higher digestible OM, NDF, and phosphorus need to be considered as explanatory factors of the relatively large difference in animal performance observed in this study.

## Conclusion

Pelleted sainfoin as a sole feed significantly reduced the population of *O. ostertagi* in young calves. Our promising results confirm the potential value of sainfoin and perhaps other tannin-rich forages with a high percentage of prodelphinidins in integrated control of bovine ostertagiosis. Although *O. ostertagi* is the main species responsible for reduced productivity in grazing calves, the apparent lack of effect against *C. oncophora* is a drawback and may hamper the practical use of sainfoin as a “broad-spectrum” anthelmintic forage. More research is needed to address the background of the lacking effect against *C. oncophora*.

## Abbreviations

AH, Anthelmintic; ALB, Albumin; CP, Crude protein; CT, Condensed tannins; DM, Dry matter; FEC, Faecal egg counts; GIN, Gastrointestinal nematodes; IP, Inorganic phosphate; OMD, Organic matter digestibility; PC, Procyanidins; PD, Prodelphinidins; PEP, Pepsinogen; SEM, Scanning electron microscopy
